# Scalable Declarative HEP Analysis Workflows for Containerised Compute Clouds

**DOI:** 10.3389/fdata.2021.661501

**Published:** 2021-05-07

**Authors:** Tibor Šimko, Lukas Alexander Heinrich, Clemens Lange, Adelina Eleonora Lintuluoto, Danika Marina MacDonell, Audrius Mečionis, Diego Rodríguez Rodríguez, Parth Shandilya, Marco Vidal García

**Affiliations:** ^1^CERN, Geneva, Switzerland; ^2^Department of Physics, University of Helsinki, Helsinki, Finland; ^3^Department of Physics & Astronomy, University of Victoria, Victoria, BC, Canada; ^4^The LNM Institute of Information Technology, Jaipur, India

**Keywords:** computational workflows, reproducibility, scalability, declarative programming, analysis preservation

## Abstract

We describe a novel approach for experimental High-Energy Physics (HEP) data analyses that is centred around the declarative rather than imperative paradigm when describing analysis computational tasks. The analysis process can be structured in the form of a Directed Acyclic Graph (DAG), where each graph vertex represents a unit of computation with its inputs and outputs, and the graph edges describe the interconnection of various computational steps. We have developed REANA, a platform for reproducible data analyses, that supports several such DAG workflow specifications. The REANA platform parses the analysis workflow and dispatches its computational steps to various supported computing backends (Kubernetes, HTCondor, Slurm). The focus on declarative rather than imperative programming enables researchers to concentrate on the problem domain at hand without having to think about implementation details such as scalable job orchestration. The declarative programming approach is further exemplified by a multi-level job cascading paradigm that was implemented in the Yadage workflow specification language. We present two recent LHC particle physics analyses, ATLAS searches for dark matter and CMS jet energy correction pipelines, where the declarative approach was successfully applied. We argue that the declarative approach to data analyses, combined with recent advancements in container technology, facilitates the portability of computational data analyses to various compute backends, enhancing the reproducibility and the knowledge preservation behind particle physics data analyses.

## 1. Introduction

**Figure 1:**
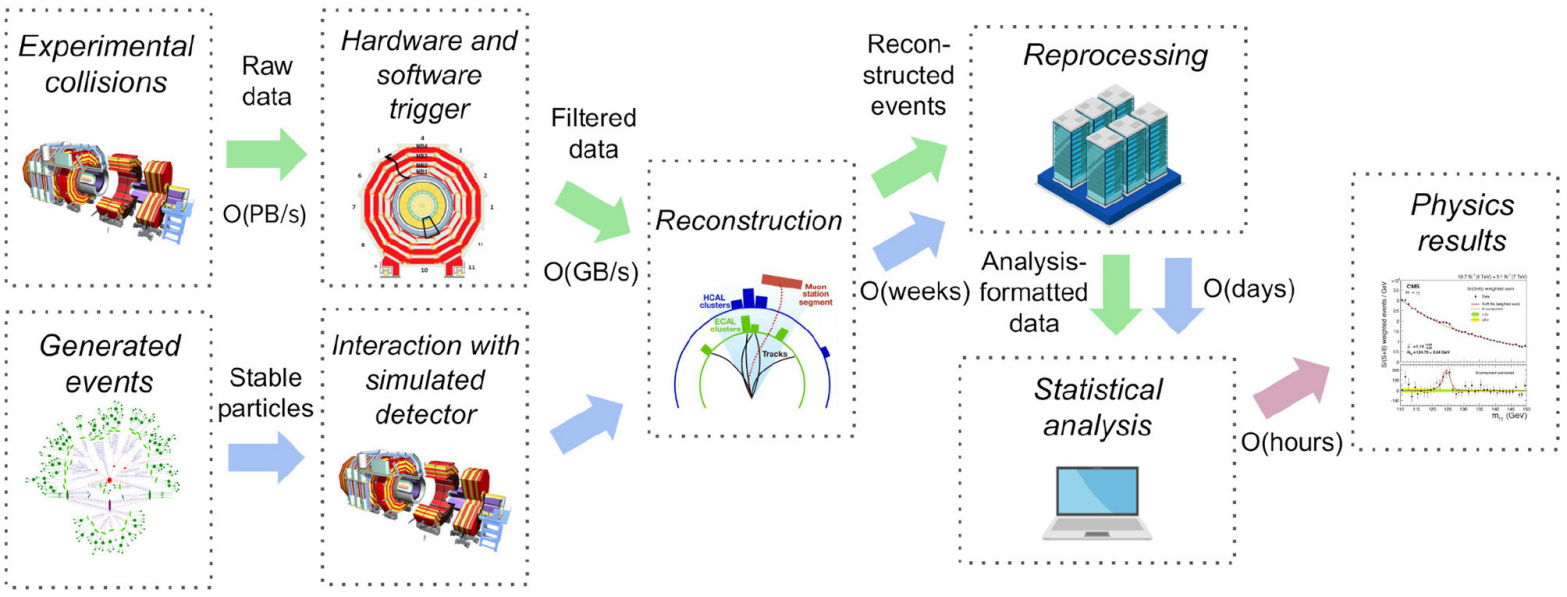
A simplified diagram illustrating typical stages in experimental particle physics data analyses. After data acquisition that is using a multi-tiered trigger filtering step, the experimental collision data are further reduced in computing processes before they are ready for physics analyses. Events generated following theoretical models undergo a detector simulation step and are subsequently subject to the same reconstruction and processing steps as the collision data. The individual analysts then compare collision and simulated data using statistical analysis techniques. Our paper focuses mostly on the computational reproducibility challenges inherent in the last data analysis stages.

Data analysis in experimental particle physics involves studying the result of particle collisions in detectors and comparing experimental results with theoretical models. The computational data processing can be roughly categorised in several stages (Albrecht et al., 2019) illustrated in Figure 1. In the data-taking stage, the data are filtered by selecting events of interest using a multi-tiered trigger system reconstructing physics objects with increasing precision. In the following processing stage, the collision data are then fully reconstructed, in many cases re-processed to profit from later improvements, and subsequently reduced into a format suitable for studying individual event signatures. Comparison to theoretical models is performed by generating events using Monte Carlo generator programs and simulating interaction with the detector. The reconstruction and later steps are the same as for the collision data. The first processing stages usually take place in big compute farms and world-wide grid computing infrastructures using automatised recipes. The processing is done by specialised teams and the end result are collision and simulated data suitable for individual particle physics analyses. The physics analysis stage uses statistical analysis techniques and is performed by individual researchers using a variety of computational approaches (Rizzi et al., 2019), from personal laptops and desktops up to small compute batch farms.

In this paper, we are focusing mostly on the latter statistical data analysis stage as performed by individual researchers. In contrast to the centralised and largely automated processing steps discussed above, the data analysis stage typically requires an iterative approach that is used to understand the data sets and optimise the overall analysis. The variety of computing approaches used, combined with a high turnover of young researchers performing the analyses in their experimental teams, poses a particular problem for computational reproducibility. The researchers typically use *imperative* programming, directly expressing all the details about the flow of necessary calculations for the compute platform at hand. This causes several challenges for possible future rerunning of the original analysis using different data, different theoretical models, updated software versions of dependent libraries, or a completely different compute backend than originally foreseen.

We argue for an alternative *declarative* data analysis approach that captures the overall knowledge associated with a particle physics analysis in a more structured way. The analysis process is expressed as a series of steps depending on other steps, each step declaring its precise sets of inputs and outputs. The structured description of the analysis process focuses first and foremost on “what” needs to be done in each step without paying particular attention to “how” the individual computation might be performed by the computer (Lloyd, 1994). This helps to design well-defined interfaces in the analysis flow, isolating unnecessary computational details until they actually matter.

We have developed a reproducible analysis platform called REANA (Šimko et al., 2019) that allows researchers to express the computational data analysis steps using such declarative paradigm. Taking advantage of recent advances of container technology in the general IT industry, the computations are isolated from supporting compute environments as much as possible. This helps with the portability of the analysis process as a whole. The REANA platform reads the structured analysis description provided by the researcher and instantiates analysis steps on containerised compute clouds. The support for various declarative workflow languages [CWL (Amstutz et al., 2016), Yadage (Cranmer and Heinrich, 2017)] and various compute backends [Kubernetes (Burns et al., 2016), HTCondor (Thain et al., 2005) for high-throughput computations, Slurm (Yoo et al., 2003) for high-performance computations] aims to ensure the universal reproducibility of computations on diverse computing platforms. The cloud-native approach of REANA, together with allowing researchers to use several high-level workflow languages or to run different parts of the same workflow on different compute backends, is what makes REANA specific when compared to other similar workflow management systems used in scientific research such as HTCondor DAGMan (HTC, 2021) or Pegasus (Deelman et al., 2015).

The paper is structured as follows. Section 2 of this paper describes the declarative approach and discusses its scalability. Section 3 demonstrates the applicability of the method on two concrete case studies from the ATLAS (ATLAS Collaboration, 2008) and CMS (CMS Collaboration, 2008) experiments analysing proton-proton collisions at the Large Hadron Collider (LHC) at CERN. Section 4 discusses wider applicability of the declarative analysis approach as well as the inherent technological and sociological challenges in the experimental particle physics domain.

## 2. Method

### 2.1. Declarative Computational Workflows

**Figure 2:**
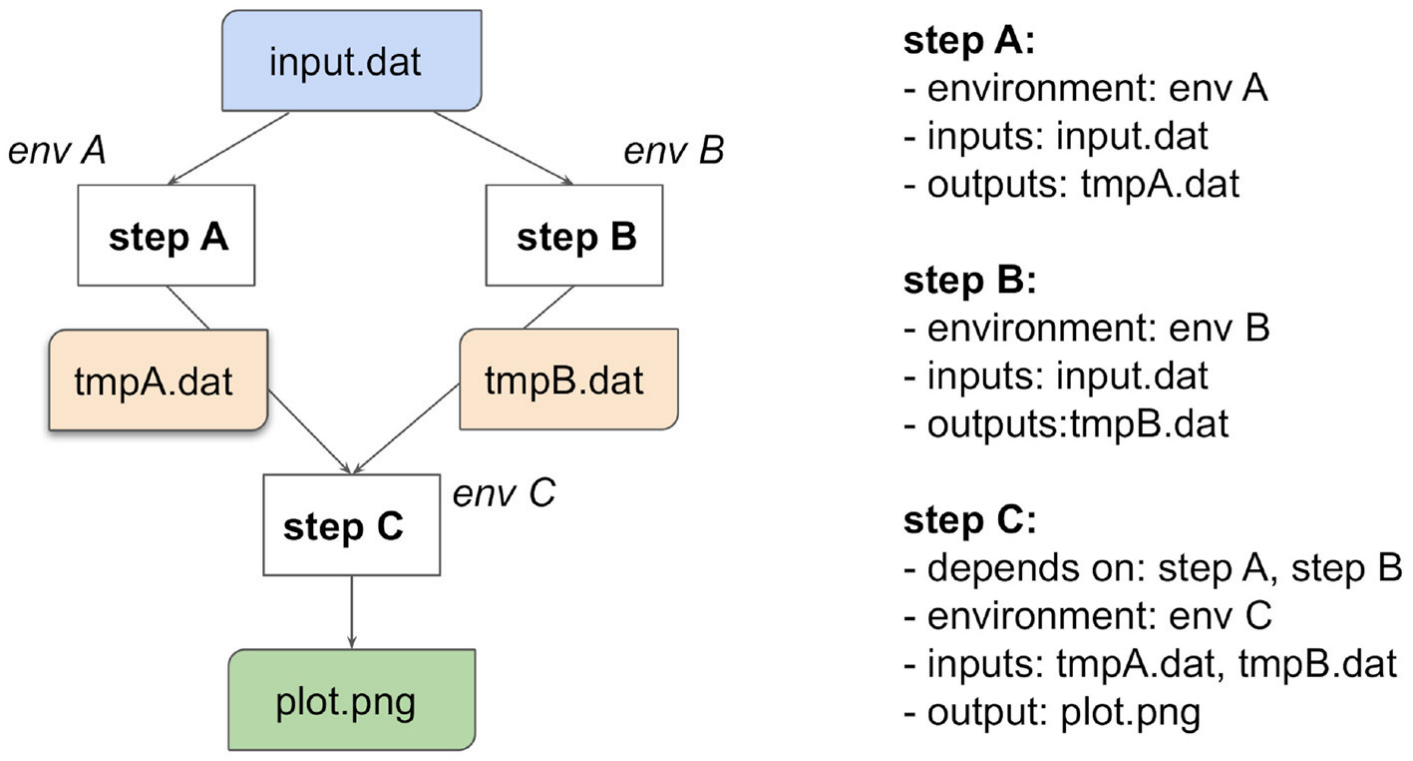
A simple computational workflow example expressed as a Directed Acyclic Graph (DAG). The left-hand side shows a graph consisting of three nodes representing computational steps A, B, and C. Note how each computational step can run in its own specific computational environment. The graph vertices represent the analysis flow. Steps A and B process the input data input.dat and can run in parallel. Step C is dependent on the temporary files tempA.dat and tempB.dat from steps A and B, respectively, and produces output plot plot.png. The right-hand side expresses the computational graph in an abstract formal language.

The computations in data analysis workflows can be represented as Directed Acyclic Graphs (DAG), where each graph vertex represents a unit of computation with its inputs and outputs and the graph edges describe the interconnection of various computational steps. Figure 2 illustrates a simple workflow composed of three steps.

There exist many formal workflow systems that allow to express the analysis graph in a structured manner (CWL, 2021). Several such systems are actively used in experimental particle physics, e.g., Snakemake (Mölder et al., 2021). One particularity of particle physics workflows is the processing of large data sets. A typical data set is several terabytes large and is composed of several thousands of files. Data processing is “embarrassingly parallel,” meaning that each file can be processed separately. Particle physics workflows are therefore suitable to generate thousands of computational steps. The analysis pipeline could consequently start tens of thousands of parallel jobs, but it would not be practical to start all the jobs at the same time. It is here where the various declarative workflow systems differ (Šimko et al., 2018) in offering more or less appropriate mechanisms for job orchestration.

One structured workflow system that is particularly suitable for deployment in these massively parallelisable scenarios is Yadage (Cranmer and Heinrich, 2017). The processing of many parallel files can be described as following the “scatter-gather” paradigm, where a workflow step processing the input data is scattered into many parallel jobs, the results of which are gathered at the end. Yadage supports a “multi-cascading scatter-gather” extension of the paradigm, which allows to scatter the input over several consecutive layers or batches of processes, optimising the parallelisation of the process for the number of available computing nodes.

**Figure 3:**
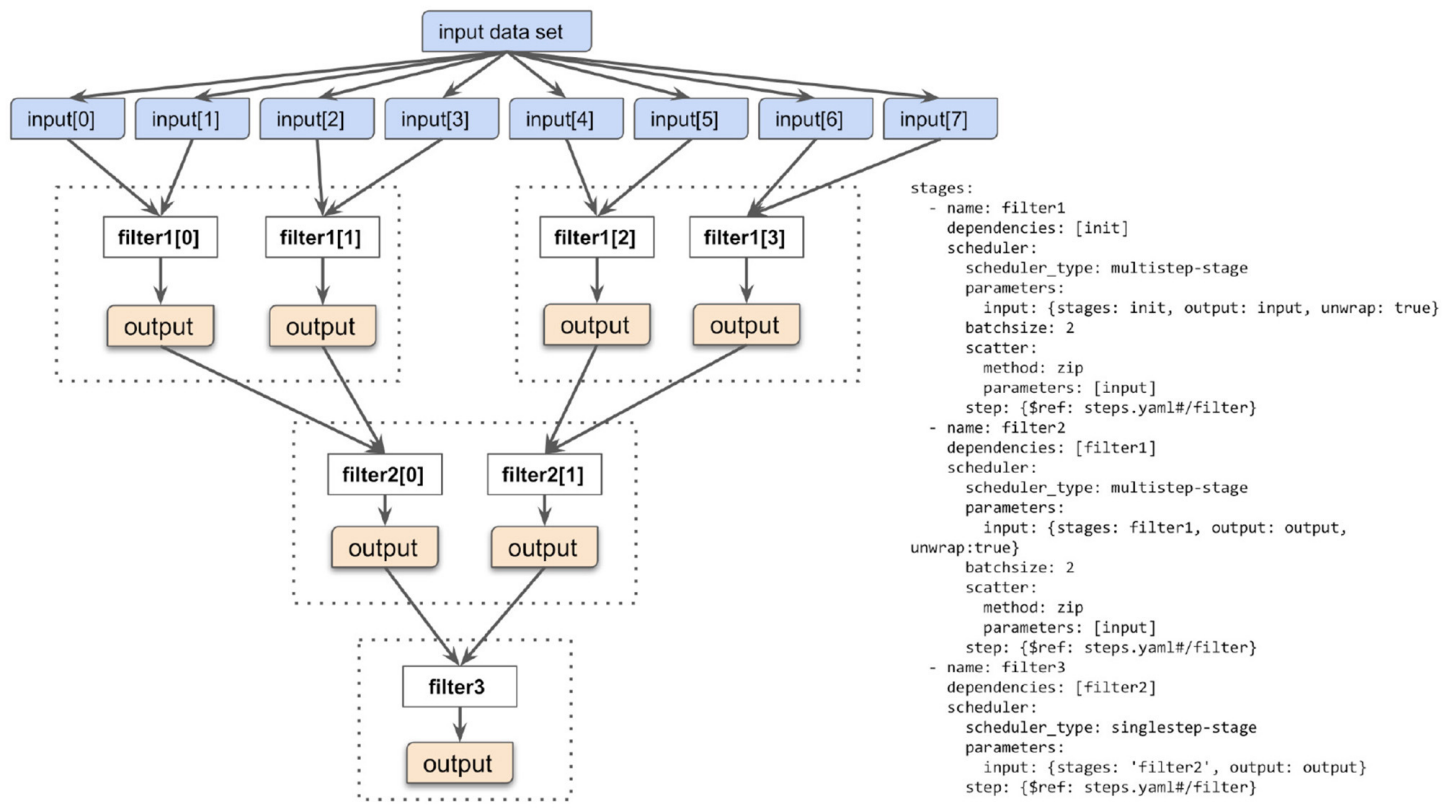
Multi-cascading scatter-gather paradigm. The input data set is composed of many files upon which the data filtering operation is to be performed. The multi-cascading scatter-gather paradigm allows to perform filtering in a cascade of stages so that the filtering operation would not process more than some desired number of input files at once. In this example, no more than two input files are processed by one filtering step. This is expressed by the notion of “batch size” in the Yadage YAML language. The batch size can be tuned depending on available processing power such as the number of processor cores.

Figure 3 presents a simple example of such a cascading workflow. The workflow applies a filtering procedure operating over the input data files in such a way that each filtering job processes no more than two data files. This is governed by the “batchsize” parameter of the Yadage specification. The workflow engine takes care of dispatching and orchestrating the necessary jobs in an automated way. This illustrates the main advantage of declarative analysis approach: the researcher can focus on describing the necessary filtering computations, and provide a hint about the reasonable number of input files to process at once. The focus is therefore on expressing “what” the filtering should be doing, without focusing on “how” the individual filtering jobs are orchestrated in a layered cascade.

### 2.2. REANA Reproducible Analysis Platform

**Figure 4:**
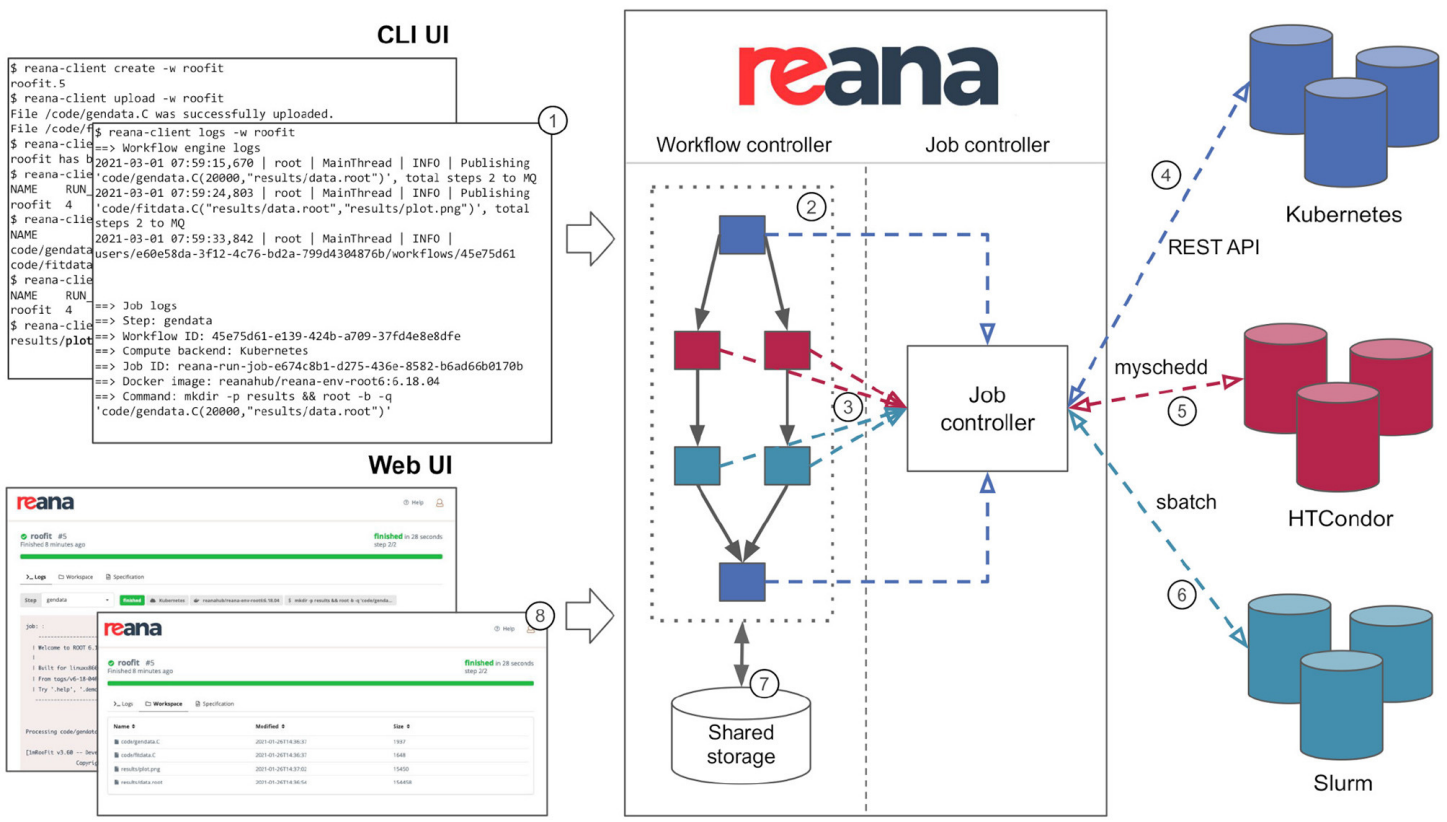
REANA reproducible analysis platform presented from a researcher's point of view. The researcher interacts with the platform by means of a command-line client (1). The researcher submits a workflow to the REANA platform for execution. REANA parses the DAG workflow (2) and for each job (3) dispatches the job execution to the desired supported computed backend such as Kubernetes (4), HTCondor (5), and Slurm (6). The temporary results of each jobs are stored on a shared file system (7). The researcher can use the web user interface (8) to inspect the status of the running workflows and to view and compare the results.

The REANA reproducible analysis platform allows researchers to use several declarative workflow systems (CWL, Serial, Yadage) and that runs analysis workflows on several supported compute backends (Kubernetes, HTCondor, Slurm). Figure 4 describes the overall architecture of the platform from the point of view of researchers. The researcher starts by describing the analysis as a series of workflow steps using structured YAML files similar to the one presented in Figure 2. Each step is described in terms of its inputs, outputs, the compute environment to use, and the command to run in order to process the output. The workflow is then submitted to the REANA platform for execution by means of a command-line client. The REANA platform parses the workflow specification and orchestrates the execution of the workflow graph by dispatching its individual jobs to the necessary compute backends. The REANA platform uses shared storage to exchange temporary data between individual jobs. The researcher can inspect the result of the workflow using a web interface.

Thanks to the declarative analysis approach, researchers can influence the workflow execution. For example, to use another computing backend, the user only has to change the compute_backend clause in the workflow step configuration, and REANA will take care of dispatching jobs as necessary. A hybrid workflow execution (Rodríguez et al., 2020), where different steps of the workflow are dispatched to different compute backends, is also supported. This allows users to use HTCondor high-throughput compute backend for processing large data sets all the while seamlessly switching to Kubernetes for producing final plots.

The scalability of the solution is ensured by the scalability of the supported compute backend platforms. The primary compute platform of REANA is Kubernetes. Each research workflow runs as a Kubernetes pod (KUB, 2020) which orchestrates the creation and deletion of individual jobs. Kubernetes is known to scale to clusters comprising many thousands of nodes. In the final stages of the analysis process, which are the focus of this study, the individual user workflows require significantly less resources than the preceding reconstruction stage. The REANA platform therefore necessitates few nodes to guarantee good response times. The scalability of the cluster depends on the number of parallel users and the number of parallel workflows that are executed at a given moment. The responsiveness of the platform can be increased by adding more nodes to the system or by creating additional clusters. For the two ATLAS and CMS use cases described in section 3, we have used REANA deployment clusters consisting of one up to 10 nodes with typically eight virtual cores and 16 gigabytes of memory per node.

### 2.3. “Continuous Analyses”

Researchers typically use a source code management system such as GitHub or GitLab for developing analysis code. These source code management platforms are often paired with associated continuous integration (CI) services, such as GitHub Actions or GitLab CI. A CI service assists software developers in ensuring the code's correctness and quality. It is therefore desirable to use CI practices for the development of structured analysis workflows themselves.

The REANA platform allows researchers to develop and debug workflows using only a subset of input files. In this way, the compute times are faster and the researcher receives prompt feedback about the status of the developed workflow. Using a test subset of input data helps to ensure workflow correctness, similarly to the CI services commonly used in software development. However, contrary to the generic CI-like use case, the REANA platform focuses on running long research computations that would otherwise time out on a generic CI system.

**Figure 5:**
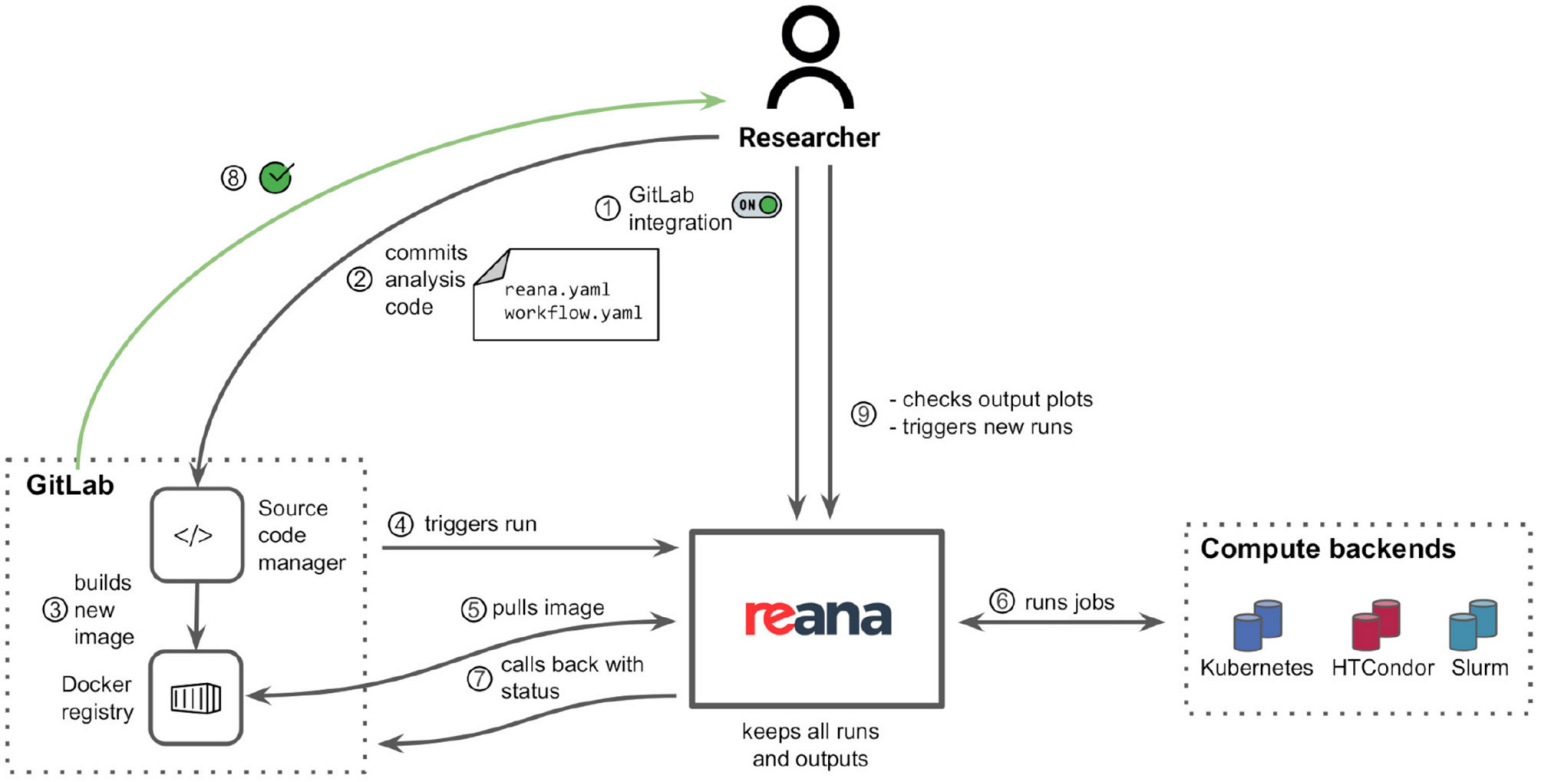
Integration of the GitLab source code management system with the REANA reproducible analysis platform. The researcher pairs the two platforms using the OAuth2 authorisation protocol (1). Afterwards, the researcher develops the workflow (2) and commits it to the GitLab system, which possibly updates the container environment images (3). The new workflow code triggers a workflow run on REANA (4), which dispatches workflow jobs to supported compute backends (5). The REANA platform informs GitLab about workflow status (7), which then alerts the researcher (8). The REANA platform keeps all the workflow run outputs that are directly accessible by the researcher (9).

We have developed a GitLab-REANA bridge (Wanderley, 2019) that allows researchers to use GitLab as the source code management platform to develop their workflows and REANA as a CI service to run them. Figure 5 illustrates the developed integration. The two platforms are brigded by means of the OAuth2 authorisation protocol (OAU, 2012). When the researcher commits changes to the workflow code in the GitLab repository, a run of the workflow on the REANA platform is triggered. As soon as the workflow run finishes, the REANA platform informs the GitLab platform about the status of the workflow run via callbacks. GitLab can then notify the researcher about the success/failure status of the workflow run or display the progress logs. Note that REANA keeps all the past runs and the produced output, allowing researchers to use the REANA web interface directly to display the outputs of a workflow run, compare different workflow runs, or trigger a new run directly.

The integration between the GitLab source code management system and the REANA reproducible analysis platform offers researchers a possibility to use all the compute backends supported by REANA, notably Kubernetes, HTCondor, and Slurm. This facilitates developing declarative analysis workflows without any technical runtime restrictions for the commonly used compute backend platforms in the particle physics community.

## 3. Results

We have modelled several typical particle physics analyses and expressed them as REANA workflows. In this way, we have studied the applicability of the containerised approach for the particle physics subject domain that usually uses large and dynamically-served software frameworks [such as CMSSW (CMS Collaboration, 2008) served via CernVM-FS (Blomer et al., 2011)]. We have expressed the analysis computations in the Yadage workflow language in order to validate the declarative programming paradigm. The following subsections describe in detail concrete studies from the ATLAS and CMS experiments studying proton-proton collisions at the CERN LHC.

### 3.1. Reinterpreting ATLAS Beyond-the-Standard-Model Analyses

There are ongoing efforts within the ATLAS collaboration to preserve analyses that search for physics beyond the standard model (BSM) so that they can be efficiently reinterpreted using alternative theoretical models that would produce similar final-state signatures in the ATLAS detector. Currently, analyses are preserved for reinterpretation within the RECAST framework (Cranmer and Heinrich, 2018) using the Yadage workflow description language. The workflows are preserved in such a way that the simulated events that would be produced at the LHC and measured by the ATLAS detector according to the model(s) of new physics used to interpret the original search can be trivially replaced in the workflow with events generated using an alternative model. The preserved workflow can thereby be re-used to produce exclusion limits for any number of alternative models of new physics, thus dramatically improving the physics reach of the original search.

**Figure 6:**
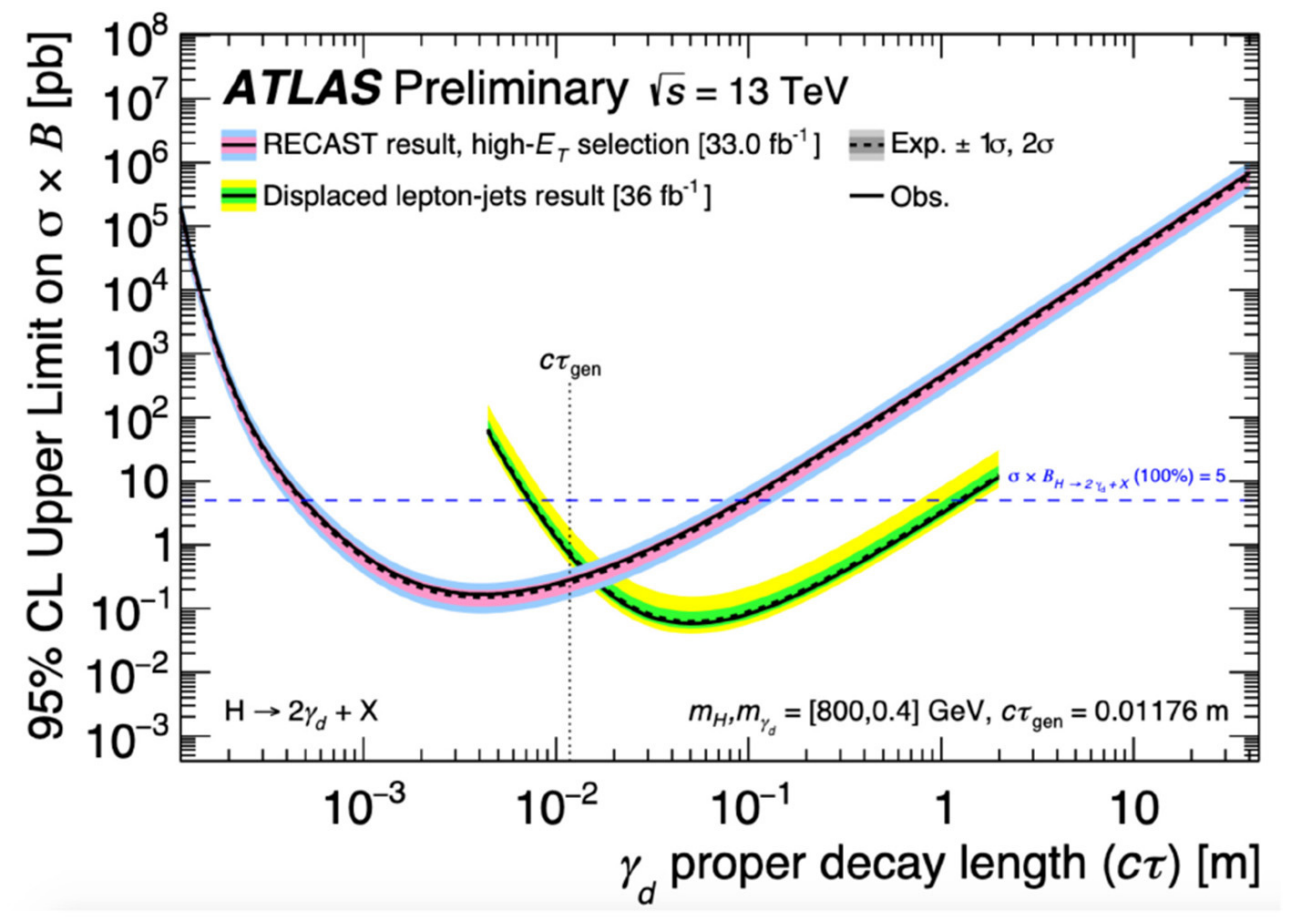
Constraints on the cross section times branching fraction of a dark photon γ_*d*_ production model obtained from the reinterpreted displaced jets search, compared with existing constraints from the original displaced lepton-jets search (ATLAS Collaboration, 2019b). These are displayed as a function of the measured distance, or “γ_*d*_ proper decay length,” that the dark photon would on average travel in the ATLAS detector before decaying. For a given decay length, cross section times branching fraction values above a given constraint curve are ruled out at 95% confidence level. Existing constraints placed by the displaced lepton-jets (the reinterpreted displaced jets) search are shown with green and yellow (pink and blue) bands representing ±1 and ±2 standard deviations of experimental uncertainty, respectively (ATLAS Collaboration, 2020).

Reinterpretations of ATLAS analyses have previously been performed to produce either novel limits on new physics models, or to extend the phase space probed by previous dedicated searches. A reinterpretation (ATLAS Collaboration, 2019a) of the search for dark matter production in association with a Higgs boson decaying to $\text{b}\bar{\text{b}}$ (ATLAS Collaboration, 2018) in the ATLAS detector was published in 2019. The reinterpreted search set novel exclusion limits on a recently-hypothesised dark matter production model (Duerr et al., 2017), in which a dark matter pair is produced via a Z' mediator in association with the emission of a dark Higgs boson, which subsequently decays to a standard model pair of $\text{b}\bar{\text{b}}$ quarks. More recently, a search for long-lived particles (ATLAS Collaboration, 2019b) was reinterpreted (ATLAS Collaboration, 2020) to set limits on three alternative models of new physics final states that would exhibit the same signature of displaced jets in the ATLAS hadronic calorimeter as was used for the original long-lived particle search. Figure 6 compares limits set by the reinterpreted displaced jets search on the cross section of a dark photon production model with limits from a dedicated lepton-jets search that considered the same model, showing that the reinterpreted analysis was able to probe new phase space uncovered by the existing dedicated search.

As REANA supports the Yadage workflow syntax used in ATLAS reinterpretations, the execution of these workflows can be readily transitioned from a traditional computing infrastructure such as bare metal and virtual machines to the REANA platform. The reinterpretation of the ATLAS “MET+jet” search, presented in this paper, is the first to have been successfully transitioned to run on the REANA platform. This search targets events containing an energetic jet along with large missing transverse momentum in the detector, and searches for an excess of such events over the background of standard model processes which exhibit the same final-state signature. The MET+jet signature is predicted in many models involving BSM physics, including supersymmetric extensions of the standard model, theories involving large extra spatial dimensions (Arkani-Hamed et al., 1998), and scenarios with axion-like particles (Mimasu and Sanz, 2015). As a result, this search is an excellent candidate for reinterpretations with new BSM models.

**Figure 7:**
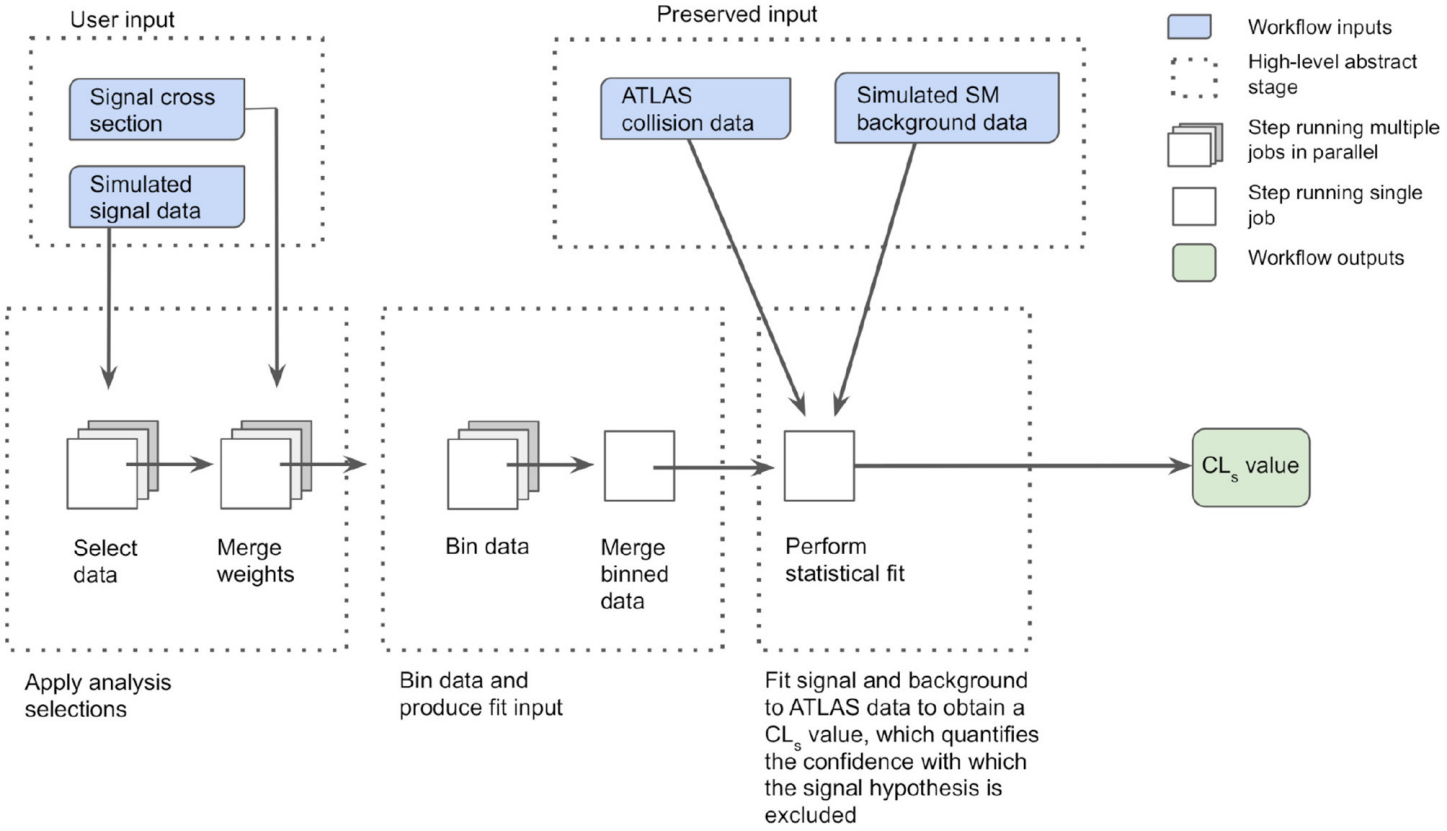
High-level overview of the ATLAS reinterpretation computational workflow for the MET+jet search. The workflow receives as an input the simulated “signal” data that would be produced in the ATLAS detector for a hypothesised model of new physics, along with the production cross section of the model. Data selections are applied, simulation weights are merged and data binning is performed in three stages. Each stage is executed as three parallel jobs. The binned data from each job is subsequently merged and forms an input to the statistical analysis framework. The framework compares the signal and standard model background data with the ATLAS collision data and computes a CL_s_ value quantifying the confidence with which the hypothesised model is excluded by the observed agreement between ATLAS collision data and the simulated standard model background data.

The MET+jet workflow, shown schematically in Figure 7, receives so-called “signal data” files as input. These contain kinematic information of final-state objects that would be measured by the ATLAS detector for simulated LHC proton-proton collision events whose decay process is described by the model of new physics under consideration. The number of such simulated events typically ranges from ~10,000 to 100,000. Additional details about the physics model, such as its predicted production cross section, are also input along with the simulated data. The workflow applies the data selections used by the search to maximise its sensitivity to the MET+jet final state of interest, and the kinematic information of interest for events passing these selections is saved to intermediate files. This initial “skimming” stage is executed in three parallel jobs—one for each ATLAS “data-taking epoch” (2015–16, 2017, and 2018)—of ~5–20 h duration. In the subsequent “weight-merging” and “data-binning” stages, weight factors associated with the simulation are merged together for each event, and the events are binned into several orthogonal analysis regions on the basis of their kinematic properties. These two stages also execute in parallel over the three data-taking epochs, and run for ~1 h. The next stage, which is executed as a single job with a run time of several seconds, merges the data from the three epochs into a single binned data set. The final stage, a single job of ~1 h duration, receives as external input simulated data describing the standard model “background” events as well as the “collision” data measured by the ATLAS detector, both of which have had the same selections applied and are binned in the same manner as the signal data. This job passes these external background and collision data sets, along with the signal data set received from the previous stage, through the statistical analysis framework. The signal and background data sets are fit to the collision data to compute a “CL_s_ value” (Read, 2002), which quantifies the confidence with which the hypothesised model of new physics can be excluded based on the observed agreement between the ATLAS collision data and the simulated standard model background data.

### 3.2. Deriving Jet Energy Corrections for the CMS Experiment

**Figure 8:**
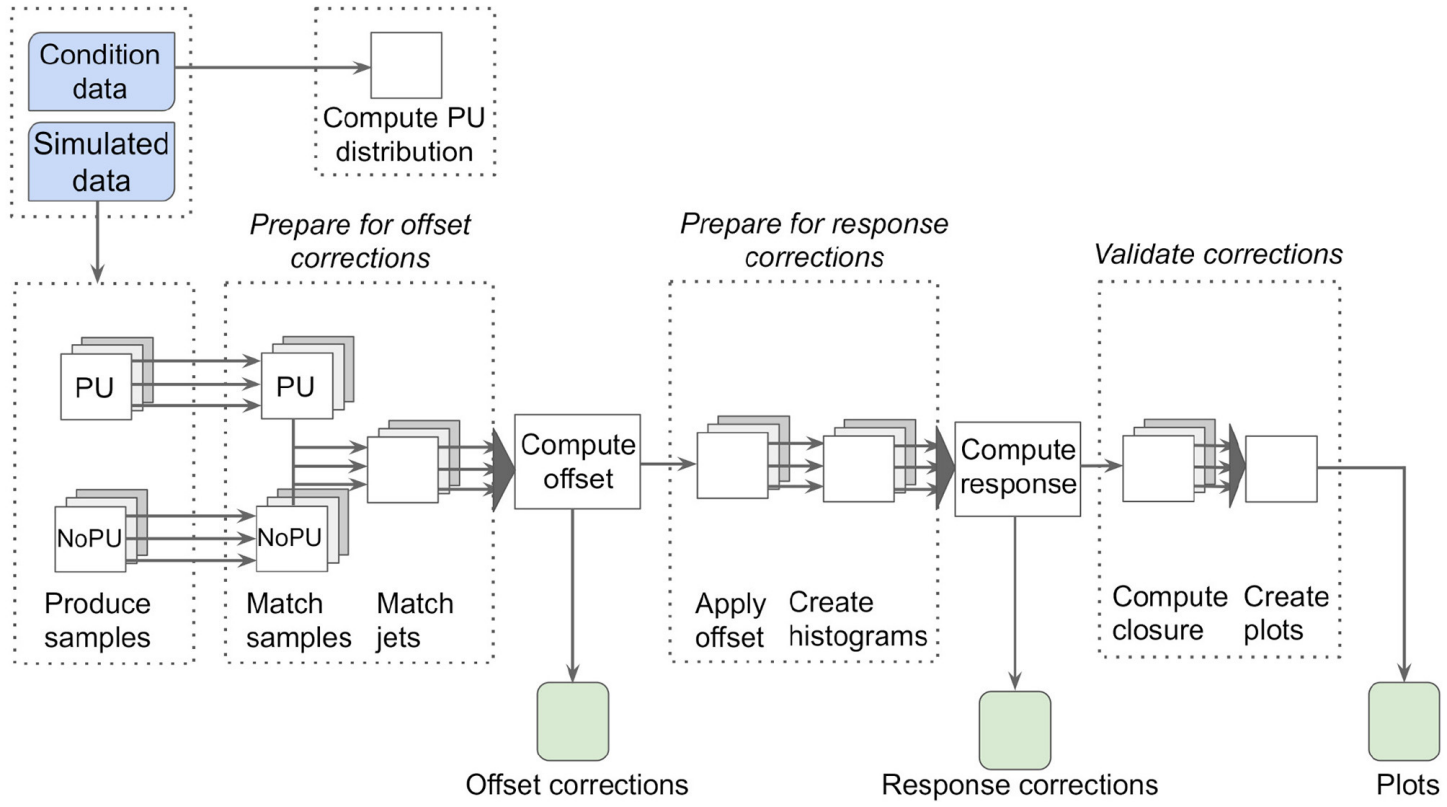
Schematic chart of the CMS offset and simulated response jet energy corrections workflow. Using simulated samples with and without pileup (PU) overlaid, a matching between particle- and reconstruction-level jets is performed to derive so-called offset corrections. After applying those corrections, response corrections are calculated. The overall procedure is then validated through a closure test applying both corrections.

The REANA platform is also suitable to re-run existing analyses on both new data and simulation samples. This is particularly useful for calibration workflows, which need to be executed on a regular basis. Typically, the overall calibration procedure remains the same so that no code modifications are required. However, the underlying CMS software release might have to be updated, e.g., to profit from improved underlying reconstruction algorithms. This can be achieved independently of REANA when having the analysis code under version control, using CI to ensure that no breaking changes are introduced. The updated container image(s) can then be executed using REANA. Further changes to reflect altered data-taking and simulation conditions can be performed entirely by modifying the REANA workflow configuration files in YAML format. The workflow can be triggered as soon as the respective input data sets are available. As an example, the first two steps of the jet energy corrections procedure performed for the CMS experiment (CMS Collaboration, 2017a), the so-called *pileup offset* and the *simulated response corrections*, are discussed in the following. The corresponding schematic chart of the workflow is shown in Figure 8.

Pileup offset corrections account for the effects of proton-proton collisions occurring within the same, previous, and subsequent bunch-crossings as the collision of interest. These additional collisions, referred to as pileup, lead to additional tracks in the tracking detectors and deposit energy in the calorimeters. They thus affect the jet energy scale and reduce the resolution of the jets associated with the collision of interest. Even though pileup is largely mitigated using various techniques (CMS Collaboration, 2020b), an offset between events simulated with and without pileup remains. In simulation, the particle level offset caused by pileup is computed as the average difference in transverse momentum between matched jets in samples with and without pileup overlay. The offset is subsequently parameterised as a function of several different observables such as the respective jet's pseudorapidity (a spatial coordinate describing the angle of the jet relative to the beam axis) to compute correction functions. Having derived the correction function parameters, the corrections are applied to the jets with pileup overlay to largely remove residual effects of pileup.

**Figure 9:**
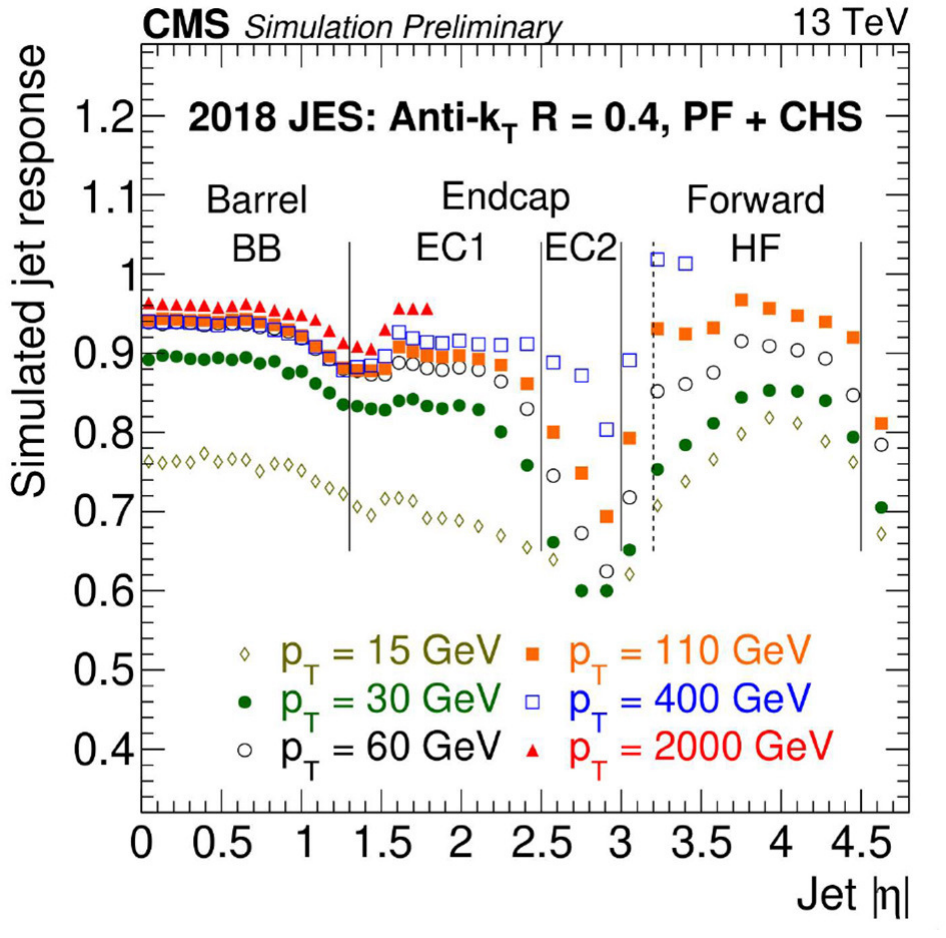
CMS simulated jet response as a function of jet absolute pseudorapidity |η| for different values of jet transverse momentum *p*_*T*_ for a given jet algorithm used in 2018. Different detector regions are indicated and separated by vertical lines. Based on these results, correction functions are derived to obtain an overall response equal to one (CMS Collaboration, 2020a).

Then the second correction step, which accounts for the simulated detector response, is performed. To derive the response corrections, jets reconstructed at particle level are compared to jets reconstructed from the particles reconstructed by the particle-flow algorithm (CMS Collaboration, 2017b), which are matched to each other for a given event employing a geometric measure. The transverse momentum ratio of reconstruction- to particle-level jets is referred to as the response and is shown in Figure 9 as an example using simulation of 2018 data-taking conditions. Correction functions are derived as functions of the jets' transverse momenta and pseudorapidity values. Finally, both the pileup offset and simulated response corrections are applied to the original jets simulated with pileup overlay to ensure closure of the method.

The main input to the calibration workflow is a large simulation sample of multijet production, which is simulated and reconstructed with and without additional collisions overlaid. This sample typically consists of tens of millions of events. The first step of the workflow consists of matching the jets reconstructed at particle level to jets reconstructed from the particles reconstructed by the particle-flow algorithm employing a geometric measure for both the samples with and without pileup. These samples are in the following denoted as *PU* and *NoPU*, respectively. In this step, the data sets are furthermore filtered to only contain information relevant for the downstream analysis and converted to a simplified data format, which enables faster analysis. The processing of this step is typically split into several hundred parallel computing jobs of several hours each and results in data sets with a total size of a few hundred gigabytes. This step has been run on the worldwide LHC grid computing infrastructure, with parallel tests running the same jobs using REANA's HTCondor integration. In the second processing step, which can again be parallelised, the corresponding jets from the “PU” and “NoPU” samples are combined to calculate the offset and resulting correction functions as discussed above. The combined data set is of the size of a few gigabytes and the files containing the subsequently resulting corrections and control distributions (histograms) are of the order of megabytes. The data set sizes for the derivation of the simulated response corrections are similar.

## 4. Discussion

The declarative analysis paradigm was successfully applied to both case studies, the ATLAS reinterpretation searches (see section 3.1) and the CMS jet energy corrections (see section 3.2). The computational workflows were described in the Yadage language and successfully executed on the REANA reproducible analysis platform. The computational environment was composed of ATLAS and CMS software stacks encapsulated as software containers.

In the ATLAS use case, several advantages over traditional computing workflow techniques were observed. The workflow description and containerisation made it possible to preserve searches for physics beyond the standard model in such a way that they could be re-interpreted with alternative models for new physics by new analysts who may have limited knowledge of how the original analysis workflow was implemented computationally. The REANA platform provided workflow developers with a centralised ready-made system dedicated for executing containerised workflows. The platform facilitated the re-running of workflows, kept track of previous runs and provided debugging tools allowing to restart part of the past workflows during the development process. Furthermore, the flexibility with which the computing resources can be allocated by REANA made it straightforward for researchers to scale up their workflows to process more simulated events with a finer grid of model parameters.

In the CMS use case, the application of the declarative workflow paradigm on the typical object calibration procedures such as the jet energy calibration workflow discussed above greatly reduced the time needed to obtain the results while reducing the workload of the analysts. The previous solution required coordination between different analysts, which may be time consuming. The containerised workflow solution presented in this paper is fully automated and can be started as soon as the input data sets are available and the workflow runs to completion without waiting for further user input. Not counting the first step, which had been run on the grid as described above, the time to obtain the results has been consequently reduced from 2 to 3 days, which includes availability of the analysts, waiting time and manual resubmission of failed jobs, to a few hours, which means that a first pass of the calibration results is available almost immediately. This provides important feedback on the data set quality to the CMS collaboration. The analysts can change and restart workflows at any stage, for example to take into account an improved parameterisation. The restarted workflows are versioned and do not overwrite previous attempts, conserving the full provenance of results and allowing detailed comparisons between various runs. A further observation inherent in using fully automated workflows was the increased analysts' time spent on improving the methodology to solve the problem at hand instead of having to run and repeat largely technical and tedious parts of work.

The use cases provided interesting input to further improvements of the REANA platform. The analysts typically run a given workflow many times with altered conditions, either to fix a problem or to study the results with altered parameters. Whilst the analyst can rerun previously finished or failed workflows with modified code or parameters, this includes instructing the REANA platform manually about the desired starting step of the desired workflow. A more automated workflow execution cache, taking advantage of full history of previous workflow runs, would memoise the CPU-intensive jobs from previous workflow runs and reuse them for new runs. This would simplify the workflow development during the active debugging phase or would allow to launch the same workflow with a different set of input parameters from the same starting point. Consequently, one would obtain the results faster.

The use cases also allowed to identify further improvements, notably more resilience in the integration with traditional external computing platforms (HTCondor) or desired integration with the worldwide LHC grid computing. Currently, if a computational step of a workflow running on HTCondor fails due to temporary unavailability of the input files, the job fails and the whole workflow execution stops, signalling an error to the user. A more robust retry mechanism, allowing analysts to specify alternative actions in case of job errors, would result in even less manual work and higher degrees of automation.

## 5. Conclusions

The present work allowed us to observe the applicability of the declarative analysis paradigm, as opposed to the more classically used imperative analysis paradigm, for two concrete ATLAS and CMS particle physics analysis use cases. The challenges were of both technological and sociological nature.

The technological questions were two-fold. Firstly, we investigated whether the traditional LHC experiment software stacks could be effectively represented in the form of software containers. Particle physics software stacks are large. Encapsulating all their dependencies may result in container image sizes of sometimes more than 20 gigabytes. It was not clear whether the mainstream container technology platforms can handle containers of that size effectively, for example the overhead related to pulling the same image onto many compute nodes. We did not observe issues with the creation of these large container images and storing them on either the Docker Hub (DOC, 2021) or the CERN GitLab registries. Further, the pulling of containers can be greatly reduced by making use of “lazy pulling”, an approach through which only those parts of the image actually required for the execution of the workload are downloaded (Mosciatti et al., 2021). Secondly, we evaluated a more general point of whether it is possible (and advantageous) to express the complex computational processes inherent in experimental particle physics data analyses in the form of declarative workflow languages, abstracting the details of the control flow. The present work provides positive answers to both technological challenges.

The sociological challenges may seem bigger. The declarative approach is less widespread than imperative approach in the particle physics data analysis landscape. Declarative programming forces the analyst to think differently about the problem at hand. The present work showed the advantage of this paradigm shift, allowing analysts to focus on the scientific methodology rather than the technical job orchestration parts of the problem.

The present work offers an indication that robust computing tools built together by computer scientists and physicists, taking advantage of the recent advances of the container technologies in the IT industry at large, provide a good basis for wider adoption and ultimate success of declarative programming approach in particle physics data analyses.

## Data Availability Statement

The data analysed in this study is subject to the following licenses/restrictions: REANA is free, open-source software published under the MIT license and is available online at: https://www.reana.io/. ATLAS and CMS datasets used in this study are restricted to the members of ATLAS and CMS collaborations. The workflows developed for ATLAS and CMS use cases are available on demand. Requests to access these datasets should be directed to ATLAS: Danika Marina MacDonell, danikam1@uvic.ca; CMS: Clemens Lange, clemens.lange@cern.ch.

## Author Contributions

AM, DR, MV, PS, and TŠ contributed especially to the REANA reproducible analysis platform development and operations. DM and LH contributed especially to the ATLAS reinterpretation workflow use case. AL and CL contributed especially to the CMS jet energy correction workflow use case. All authors took part in preparing the containerised declarative workflow concept and contributed to its realisation.

## Conflict of Interest

The authors declare that the research was conducted in the absence of any commercial or financial relationships that could be construed as a potential conflict of interest.
